# A Survey on Blood Pressure Measurement Technologies: Addressing Potential Sources of Bias

**DOI:** 10.3390/s24061730

**Published:** 2024-03-07

**Authors:** Seyedeh Somayyeh Mousavi, Matthew A. Reyna, Gari D. Clifford, Reza Sameni

**Affiliations:** 1 Department of Biomedical Informatics, Emory University, Atlanta, GA 30322, USA; seyedeh.somayyeh.mousavi@emory.edu (S.S.M.); matthew.a.reyna@emory.edu (M.A.R.); gari@dbmi.emory.edu (G.D.C.); 2 Biomedical Engineering Department, Georgia Institute of Technology, Atlanta, GA 30332, USA

**Keywords:** blood pressure, cuff-based blood pressure, bias in blood pressure, machine learning, individualized medicine, demographics

## Abstract

Regular blood pressure (BP) monitoring in clinical and ambulatory settings plays a crucial role in the prevention, diagnosis, treatment, and management of cardiovascular diseases. Recently, the widespread adoption of ambulatory BP measurement devices has been predominantly driven by the increased prevalence of hypertension and its associated risks and clinical conditions. Recent guidelines advocate for regular BP monitoring as part of regular clinical visits or even at home. This increased utilization of BP measurement technologies has raised significant concerns regarding the accuracy of reported BP values across settings. In this survey, which focuses mainly on cuff-based BP monitoring technologies, we highlight how BP measurements can demonstrate substantial biases and variances due to factors such as measurement and device errors, demographics, and body habitus. With these inherent biases, the development of a new generation of cuff-based BP devices that use artificial intelligence (AI) has significant potential. We present future avenues where AI-assisted technologies can leverage the extensive clinical literature on BP-related studies together with the large collections of BP records available in electronic health records. These resources can be combined with machine learning approaches, including deep learning and Bayesian inference, to remove BP measurement biases and provide individualized BP-related cardiovascular risk indexes.

## 1. Introduction

In 2021, the World Health Organization (WHO) reported that 32% of the world’s mortality is related to cardiovascular diseases (CVDs) [1]. In 2020, CVDs were the leading cause of death in the United States, surpassing cancer and COVID-19 [2]. Strokes and heart attacks are the leading causes of CVD-related mortalities [1,3,4], and hypertension is the most significant risk factor for CVDs [5,6,7]. Hypertension rarely shows early symptoms before causing severe damage to organs such as the heart, blood vessels, brain, eyes, and kidneys [8]. Therefore, it is known as the “silent killer” [9,10]. Monitoring blood pressure (BP) is one of the most effective and widely accessible methods for diagnosing and reducing CVD prevalence [11]. Abnormal BP is even more critical and life-endangering for vulnerable populations, including the elderly and pregnant women.

BP is measured manually and automatically in medical centers. Most commercial BP devices use a *cuff*—a non-elastic fabric commonly wrapped around the arm—to apply sufficient external pressure on the artery wall to temporarily block the blood flow and to measure the BP when the arterial BP and the monitored external pressure are balanced. Over the decades, cuff-based BP devices have evolved from manual mercury-based devices to the current ones based on pressure sensors and automatic electronic measurements [12].

Cuff-based BP devices have passed the test of time due to their simple operation, low cost, availability, and ease of interpretation [13]. Automatic and portable BP devices have also enabled out-of-clinic ambulatory BP monitoring by patients and their families. Although ambulatory BP is not always as accurate as in-clinic measurements, it can be acquired more frequently, thereby reducing clinic visits and costs, increasing patient satisfaction, and overcoming clinical environment stress, which can lead to the so-called *white-coat* hypertension [14,15]. Specifically, the latest guidelines advise patients with gestational and chronic hypertension to repeat BP measuring at home [14].

Hypertension diagnosis and the treatment of many other diseases are based on the accurate measurement of BP. It is, therefore, critical to assess the accuracy of BP values reported in ambulatory and in-clinic settings [16,17]. However, most users are unaware or neglectful of the standard BP measurement protocols that should be followed during BP acquisition to acquire accurate BP values. Therefore, BP measurements—even in clinical settings—can be significantly biased and variable due to the measurement circumstances (beyond the patient’s physiological factors). This results in misinterpretations of BP readings and hampers the reliability of this vital sign for clinical diagnosis.

In recent years, many studies have focused on the notion of bias and its significance in different areas of biomedical research, including bias in false beliefs about biological differences between various races [18], pain assessment and treatment recommendations [19], medical equipment [20], racial biases in algorithmic diagnosis [21], performance metrics in algorithmic diagnosis [22], and reducing bias in machine learning (ML) for medical applications [23].

In this survey, we focus on the potential sources of bias in BP measurement, which can influence BP-based diagnoses of hypertensive and hypotensive patients. We focus on the most popular commercial cuff-based devices, which are currently the most accurate and popular BP devices used for in- and out-patients. They are also used for the calibration of cuff-less BP devices. We have conducted a broad literature survey in terms of the various factors that can potentially impact BP measurements, including patient-related factors, BP acquisition session circumstances, and device-related factors.

It is important to note that the objective of this work is not to conduct a systematic review, as the notion of bias is very broad and beyond the scope of a single survey article. Instead, this survey is designed to investigate studies that address bias-related aspects, both directly and indirectly. To the best of our knowledge, although certain surveys have systematically reviewed specific sources of bias in BP measurement, there has not yet been a comprehensive survey broadly encompassing various potential sources of bias in BP measurement. Our goal is to bridge this gap in the literature. This paper is organized as follows. Section 2 reviews the biophysics of BP. Section 3 classifies BP measurement methods. Section 4 investigates different validation standards and reviews various commercial BP technologies and their operating principles. Section 5 presents potential sources of bias in BP technologies from different perspectives. Section 7 details future perspectives for using machine learning techniques for individualized BP assessment and bias correction. Section 8 concludes this study and discusses its impact and limitations.

## 2. A Review of Blood Pressure Physiology

Vital signs and physiological measurements serve as proxies for assessing fundamental body functions. Together with other vital signs, BP is one of the key clinical parameters obtained from the body [24]. A regulated BP guarantees a timely and adequate supply of blood [25], which is essential for the following blood functions: (1) transportation of nutrients, waste, hormones, oxygen, and carbon dioxide; (2) regulation of osmotic pressures, temperature, and pH; and (3) protection against infections via white blood cells, antibodies, and blood clotting to prevent excessive blood loss during injuries.

### 2.1. Blood Pressure Definition

**Figure 1:**
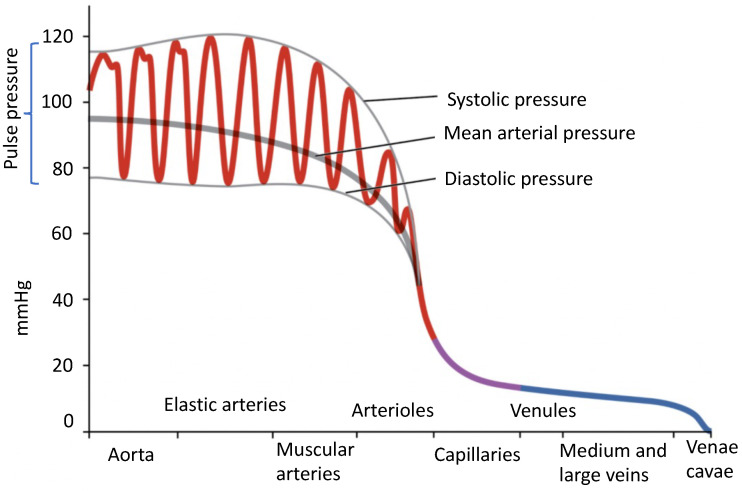
The systolic, diastolic, mean arterial, pulse pressure, and overall blood pressure at different blood vessel points; adopted from [28] by OpenStax College (CC-BY-3.0). The color scheme acts as a visual guide, highlighting blood oxygenation levels and flow directions. The use of red symbolizes freshly oxygenated blood from the lungs, coursing through arteries to nourish body tissues. A gradual shift to blue indicates decreasing oxygen levels, signifying an increase in deoxygenated blood. Blue represents deoxygenated blood returning to the heart through veins after delivering oxygen to tissues.

**Figure 2:**
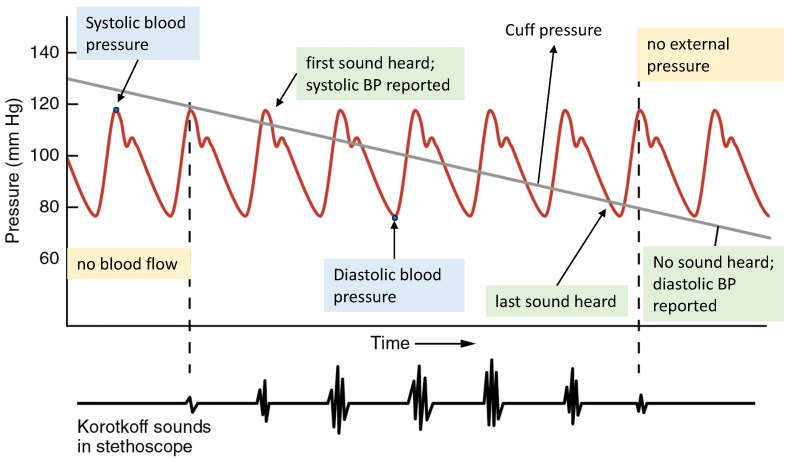
The auscultatory method measures blood pressure based on Korotkoff sounds. Note the difference between the actual and reported systolic/diastolic blood pressures; adapted from [32] (CC-BY-3.0).

Blood flows throughout the body due to the pressure difference in the arterial system [26]. BP assesses the mechanical function of the heart, acting as a pump. It is the force per unit area of the arterial system, commonly measured in millimeters of mercury (mmHg). In healthy subjects, the heart contracts between 60 and 100 times per minute, resulting in a pulsatile and almost periodic pressure wave in the arterial system. The pressure wave’s maximum occurs during *systole* when cardiac contraction exerts its maximum pressure on the blood and arterial walls. This is when blood is pumped from the heart into the arteries. The lowest pressure corresponds to *diastole*, when the myocardium relaxes, allowing the chambers to refill with blood [27]. Therefore, arterial BP fluctuates between the maximum and minimum levels and gradually decreases to zero as it reaches the end of the circulatory system, as shown in Figure 1 [28]. Throughout this work, we study the following BP parameters:

- *Systolic blood pressure (SBP)*, which represents the maximum pressure inside the arteries during cardiac contraction;
- *Diastolic blood pressure (DBP)*, which represents the minimum arterial pressure during cardiac rest;
- *Mean Arterial Pressure (MAP)*, an empirical weighted average of SBP and DBP used to approximate the average BP over the cardiac cycle using only its maximum and minimum values [29]:
  (1) $$\mathrm{MAP}=\frac{\mathrm{SBP}+2\times \mathrm{DBP}}{3}$$
  where the higher weight of the DBP empirically accounts for the asymmetry of the continuous BP (see Figure 2).

### 2.2. Factors Impacting Blood Pressure

Arterial BP values are subjective and depend on many factors, including the physics and physiology of the body; body position; brain activities; digestive activities; muscle activities; nerval stimulations; environmental factors (air temperature and audio noise level); smoking; alcohol and coffee consumption; and medications [30,31]. There are also various biological factors that impact BP [32], as outlined below.

#### 2.2.1. Cardiac Output

Cardiac output is the amount of blood pumped into the ventricles by the heart. BP and blood flow increase when factors such as sympathetic stimulation, epinephrine and norepinephrine, thyroid hormones, and increased calcium ion levels increase cardiac output (heart rate, stroke volume, or both). Conversely, factors that decrease heart rate, stroke volume, or both, such as parasympathetic stimulation, increased or decreased levels of potassium ions, decreased calcium levels, anoxia, and acidosis, decrease cardiac output [30,32].

#### 2.2.2. Compliance

Compliance is the ratio of the change in volume to the change in pressure applied to a vessel [33]. Arterial compliance has a direct relationship with its efficiency. There is an inverse relationship between BP and the compliance of blood vessels. When vascular diseases cause artery stiffening, compliance decreases, and the heart works harder to push blood through the stiffened arteries, resulting in an increase in BP [32].

#### 2.2.3. Blood Volume

The total amount of blood in the body directly affects blood flow and blood pressure. If blood volume decreases due to bleeding, dehydration, vomiting, severe burns, or certain medications, BP and blood flow also decrease. However, the body’s regulatory mechanisms are efficient in controlling BP, and symptoms may not appear until 10-20% of blood volume is lost. Hypovolemia can be treated with intravenous fluid replacement, but the underlying cause must also be addressed to restore homeostasis in these patients. Intravenous fluid replacement is typically part of the treatment [32,34].

#### 2.2.4. Blood Viscosity

Blood viscosity refers to the fluid’s thickness (resistance to blood flow). Blood viscosity is inversely proportional to flow and directly proportional to resistance. As a result, any factor that increases blood viscosity raises the resistance and lowers the flow. In contrast, factors that decrease viscosity increase flow and lower the resistance. Blood viscosity typically does not vary over short time intervals. Plasma proteins and the formed elements are the two primary factors influencing blood viscosity. Any condition that affects the number of plasma constituents, such as red blood cells, can change viscosity [32]. Since the liver produces most plasma proteins, liver impairments or dysfunctions such as hepatitis, cirrhosis, alcohol damage, and drug toxicity can also alter viscosity and decrease blood flow [35].

#### 2.2.5. Blood Vessel Length and Diameter

The resistance and length of vessels are directly related. Longer vessels have more resistance and lower flow. A higher surface area of the vessel makes it harder for blood to flow through. Similarly, shorter vessels have smaller resistance, resulting in higher flow. The length of blood vessels grows with age, but they tend to stabilize and remain constant in length during adulthood under normal physiological conditions [30,32]. The diameter of blood vessels differs depending on their type and can change throughout the day in response to chemical and neural signals. Unlike vessel length, vessel diameter is inversely related to resistance. Intuitively, a vessel with a larger diameter allows blood to flow with less friction and resistance (even with the same blood volume), because the blood has less contact with the vessel walls [32,36].

### 2.3. Blood Pressure Norms

In 2018, the Journal of the American Heart Association (AHA) published guidelines for the prevention, detection, evaluation, and management of BP [37]. Table 1 lists the BP ranges for adults in four categories. BP values for children and adolescents are generally lower than those for adults, and they gradually rise with age [38,39]. Hypertension (abnormally high BP) is characterized by a continuous elevation of BP values above the normal range. On the other hand, hypotension (abnormally low BP) occurs when BP values are below normal ranges. Hypotension can happen due to sudden blood loss or a decrease in blood volume, and hypertension is linked to an increased risk of various forms of CVDs [37].

## 3. Blood Pressure Measurement Methods

BP readings are also impacted by the measurement technology utilized, measurement setup, patient conditions, and time. We study these factors in the sequel to this paper.

### 3.1. Invasive Blood Pressure Measurement

In this type of measurement, a *catheter*—a thin tube—is inserted into a vessel to access blood for laboratory testing and to measure arterial blood pressure using a pressure transducer consisting of a delicate and sensitive diaphragm. The transducer’s resistance varies with the slightest pressure changes, allowing for the detection of BP fluctuations [16]. This technique is utilized to record and monitor changes in BP [40].

In modern healthcare facilities, disposable pressure transducers are commonly utilized for more precise and continuous measurement of BP in specialized settings, such as cardiac catheterization labs, intensive care units (ICUs), and operating rooms. Although pressure transducers can measure intracranial and intra-abdominal pressures, they are most frequently used for invasive monitoring of arterial and venous BP. Invasive methods of monitoring BP can be generally classified into two categories [41]: *intravascular*, where the pressure sensor is inserted into the vessel at the tip of the catheter, and *extra-vascular*, where the pressure sensor is located outside the vessel and along the catheter’s end.

### 3.2. Non-Invasive Blood Pressure Measurement

Non-invasive BP measurement techniques determine BP without any physical injury to the body. This technique is classified into two groups: cuff-based and cuff-less methods. Cuff-less methods are still in the developmental stage and are uncommon in clinical studies. Therefore, the scope of the current survey is on cuff-based methods.

All commercially available cuff-based BP measurement technologies consist of a manometer (digital or analog), a pressure pump (manual or automatic), and a cuff. The basic principle of these devices is to apply sufficient pressure to the extremities (arm, wrist, or leg) to temporarily block the blood flow through the artery. The cuff is then slowly deflated, and the pressure is reduced until the blood begins to flow through the artery. At this point, the pressure in the cuff is close to the SBP—the peak of BP (Figure 2). Since BP oscillates through the cardiac cycle, it repeatedly falls below the external pressure, which results in repeated obstruction of blood flow (which can be heard through a stethoscope or sensed via a measurement device). The cuff pressure is then further reduced until blood flows through the artery with no obstruction, where the pressure in the cuff drops below the DBP, and no further sounds are heard by the physician (or sensed by the automatic analysis hardware/software). In Figure 2, we can see how the actual and reported BP values can deviate, especially for the DBP.

There are various methods for identifying the moments when the cuff pressure is equal to the SBP and DBP. The main difference between these methods lies in their ability to accurately detect the external-internal pressure balance points [26]. These methods are outlined below.

#### 3.2.1. Auscultatory

This method dates back to the late 18th century and remains the gold standard for validating novel BP measurement methods [42]. It is based on Korotkoff sounds, which are produced by the turbulent flow of blood through the compressed artery. As the cuff pressure is slowly released, the artery begins to open, and its pressure exceeds the cuff pressure. The pressure that the manometer displays when the first Korotkoff sound is heard corresponds to the systolic BP. As the cuff pressure reduces, the blood flows turbulently in the artery, and the sound continues until the pressure of the cuff reaches the lowest arterial pressure. At this moment, the Korotkoff sound disappears, and the corresponding manometer pressure is the diastolic BP, as shown in Figure 2 [42,43].

Initially, BP was measured using mercury sphygmomanometers, but the toxicity of mercury led to the adoption of aneroid sphygmomanometers, which use a mechanical pressure gauge that is calibrated to display the pressure readings. The precision of the aneroid sphygmomanometer depends on the operator’s proficiency in auscultation, use of a stethoscope, and visual acuity [42]. More recently, hybrid sphygmomanometers have been used to automate the detection of Korotkoff sounds and the display of the SBP, DSP, and pulse rate on digital monitors [44]. Notably, aneroid sphygmomanometers have varying accuracy across manufacturers. Over the past decade, surveys have been conducted to assess the accuracy of these devices. The results demonstrate that the BP readings from different manufacturers have significant deviations ranging from 1 to 44% [42,45,46,47]. Additionally, using a small gauge to read the pressure is another potential source of bias in these devices [42]. Therefore, mercury sphygmomanometers remain popular in clinical settings.

#### 3.2.2. Oscillometric

This method is currently the most popular technology for automated BP measurement devices [13]. Using a pressure sensor, it measures the pulsatile BP in the artery during cuff inflation and deflation [11]. In this technology, the transducer detects the small variations in arterial pressure oscillations or the intra-cuff pressure, produced by the changes in pulse volume due to the heartbeats [13]. The oscillations begin when the cuff pressure exceeds the systolic BP and continue until it is lower than the diastolic BP. A microcontroller is used to control the inflating process, reading the analog output signal of the pressure sensor and its digitization. The micro-variations of the sensed pressure are filtered and pre-processed to obtain the mean arterial pressure, as defined in (1) [13]. Due to the indirect nature of this approach, the measured pressure value requires calibration to be mapped to the actual systolic and diastolic BPs. The microcontroller displays the measured BPs on a local screen [42].

Some of the advantages of the oscillometric method are as follows [13,42]: (1) ease of use for patients (placement and removal of the cuff); (2) ease of calibration (commonly via a button on the device); (3) portability; and (4) use with minimal training. The negative aspect of this method is that commercial oscillometric devices use different (and commonly proprietary) algorithms to estimate BP from the measurements [48]. This results in an intrinsic source of bias in BP measurements among devices from different manufacturers. It is important to note that although these algorithms have been validated in adult populations, there is a lack of validation in the youth population.

#### 3.2.3. Ultrasound

This technique is based on the Doppler effect [13]. Similar to the previous methods, cuff inflation blocks the blood flow in the artery. Then, as the cuff deflates, the arterial wall starts to move at the systolic BP and produces a Doppler phase shift in the reflected ultrasound. As the arterial motion decreases and reaches its endpoint, the cuff pressure at that moment is considered the diastolic BP [42]. The detection of the onset and offset of arterial wall motions is performed by processing the Doppler reflection using a local (digital) processor.

## 4. Cuff-Based Blood Pressure Measurement Technologies

As with all medical equipment, BP measurement devices must meet regulatory requirements and standards. In this section, the essential BP measurement and validation standards of commercial cuff-based BP monitors are reviewed.

### 4.1. BP Measurement Device Standards

There are different standards for validating BP measurement devices. In 1987, the Association for the Advancement of Medical Instrumentation (AAMI) published the first standard for non-invasive BP medical devices [49]. In 1990, the British Hypertension Society (BHS) established another clinical protocol for validating these devices, which included many of the AAMI standards [50,51]. These parallel standards continued until 2018 when the AAMI, the European Society of Hypertension (ECH), and the International Organization for Standardization (IOS) published a universal protocol named the “single universal standard”, also referred to as ISO 81060-2:2018/Amd 1:2020 on “non-invasive sphygmomanometers”, [13,52]. This unified standard facilitated the validation and comparison of measurements made by BP devices manufactured globally.

As part of this standard, manufacturers are required to collect a database of measurements from at least 85 individuals, with each individual being measured at least three times. Therefore, at least 255 records are required to validate BP devices [26]. Moreover, the mean absolute error between the recorded BP values and the reference technology should be less than 5 mmHg, and the standard deviation of the multiple measurements should be less than 8 mmHg. The standard also categorizes the cumulative absolute error of BP measurement devices into three groups: less than 5 mmHg, 5 mmHg to less than 10 mmHg, and 10 mmHg to less than 15 mmHg. Devices can only pass the certification test if at least 85 percent of the reported results based on the noted criteria are less than 10 mmHg [13]. The reference BP measurement technology is the invasive BP measurement technique (Section 3.1), but it is also acceptable to compare the BP results with any non-invasive measurement method with a maximum error of 1 mmHg [13].

### 4.2. Commercial Cuff-Based BP Measurement Devices

Commercial BP devices can be categorized into three groups: ambulatory BP monitors (ABPMs), office BP (OBP) monitors, and home BP monitors (HBPMs).

The ABPM is a portable monitor that is carried by individuals with hypertension (or those who are at a higher risk of developing hypertension) for a period of 24 or 48 h while engaging in their regular daily activities and during sleep. Based on the physician’s required settings, the device measures the patient’s BP at specific time intervals, e.g., 15 or 30 min. After the required period, the patient returns to the clinical center, the device is taken off the patient, and the BP data are transferred to the computer or cloud and analyzed using software [26]. ABPMs are most commonly used for detecting non-dipping BP patterns, which refer to a phenomenon where an individual’s BP fails to exhibit the normal nocturnal decrease during sleep, potentially indicating an elevated risk of cardiovascular issues [53].

OBP monitors are the most common type of BP measurement device for clinical use. Their accuracy is critical, especially in emergency and surgical units, where physicians make essential real-time decisions from the values of the BP and other vital signs. In these situations, the physician may not have adequate time to repeat BP measurement (as advised by BP reading standards). These BP devices are either integrated into bedside monitors or used as discrete devices similar to HBPMs.

### 4.3. Standard Blood Pressure Measurement Conditions

**Figure 3:**
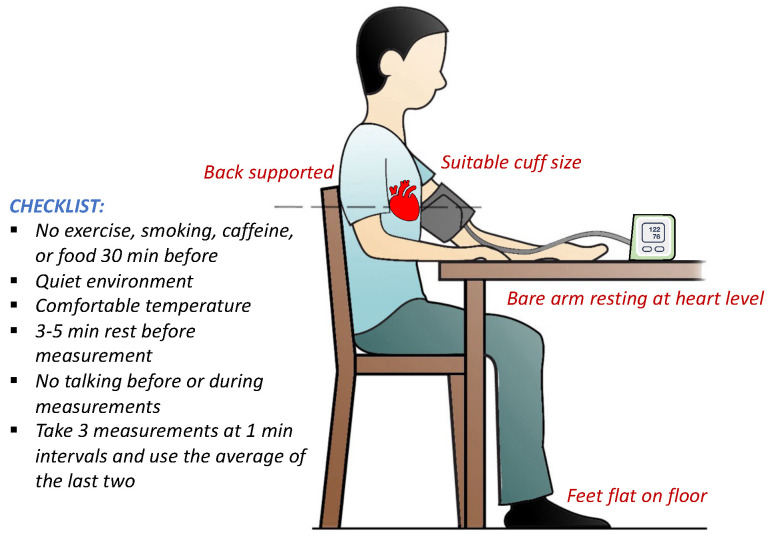
The basic principles and important standards of blood pressure measurement; adapted from [55]. See STRIDE-BP (https://stridebp.org/, accessed on 1 March 2024) for validated electronic cuff-based blood pressure devices.

Several guidelines have been published to improve the accuracy of BP measurement devices by standardizing BP acquisition procedures. Although there are different recommendations in different countries and organizations, they typically address the same fundamental issues [54]. Figure 3 illustrates the basic principles of BP measurement [55]. The following are some of the common items found in BP measurement guidelines:

- To maintain a stable BP measurement environment, it is recommended to refrain from opening and closing windows and doors. [42].
- The temperature and relative humidity of the BP measurement environment should be in the range of 15–25 °C and 20–85%, respectively [42].
- BP should be measured in a quiet environment [55,56].
- The patient should not smoke, eat, or drink for at least 30 min before measuring [55].
- The patient should have adequate rest time before the measurement to stabilize BP.
- The patient should not speak and should remain quiet during the measurement [55].
- The patient should sit on a chair with back and arm supports and without crossing their legs [56].
- The patient’s arm should be placed and remain at the same level as the heart throughout the BP measurement [42].
- An appropriate cuff should be used for measuring according to AHA guidelines (Table 2) [42].
- The antecubital fossa (the area between the anatomical arm and the forearm) should be 2-3 cm above the lower end of the cuff [57].
- During the measurement, the patient’s feet should remain flat on the floor [55].
- Measuring BP should be performed using direct contact of the cuff with the upper part of the arm (not over sleeves) [56].
- It is recommended to take three BP measurements with one-minute intervals in between. The average of the results should be reported as the BP value [55,56].

## 5. Potential Sources of Bias in Blood Pressure Technologies

Patient positions or acquisition circumstances that do not meet the measurement guidelines may potentially result in BP measurement biases (over- or under-estimation) and misdiagnosis [48,54,58]. We study the potential sources of biases under three categories [48,59].

### 5.1. Biases Related to Blood Pressure Measurement Devices

The BP measurement device is the first source of measurement bias. BP devices comprise the main measurement unit and the consumable parts, as detailed below.

#### 5.1.1. Main Blood Pressure Measurement Unit

Biases related to the main BP measurement unit are known as systematic errors. Manufacturers report the acceptable range of measurement errors in the user manuals of BP measurement devices. Measurements falling outside of this range are considered unacceptable, indicating that the device needs calibration. Therefore, they require maintenance and regular calibration to identify and reduce measurement uncertainties to an acceptable level. Medical instruments comprise numerous electro-mechanical elements that undergo natural aging and wear and are impacted by microscopic airborne contaminants that accumulate on their sensitive electronic elements and sensors. These effects change the electro-mechanical characteristics of the devices, even if they are not used, resulting in a gradual drift from their nominal operating point. The deviations of the device elements contribute to measurement biases at the system level. It should be highlighted that the effects of device aging are not the same as the failure of the elements or the device. Therefore, even though a BP device may appear new or be fully functional, it may deviate from the original calibration point. Hence, the regular calibration of medical devices is an essential requirement that should not be compromised. It is important to note that systematic errors, such as changes in the cuff deflation rate [60], are not mitigated by averaging, as they tend to introduce a constant bias into the reported values (unknown to the user) [61]. The identification and correction of systematic errors are performed using reference instruments that have been well maintained and calibrated by medical instrumentation experts.

Another source of systematic error arises from the software/algorithm used to calculate BP from the pressure sensor measurements. The lack of calibration for the electronic pressure sensors can result in systematic errors [61,62]. For example, in automatic BP measurement devices, the rate of cuff inflation and deflation is significant because the isometric exercise involved in inflating the cuff causes a temporary elevation of about 10 mmHg. Although this only takes around 20 s, if the cuff is deflated too quickly, the pressure may not have returned to baseline, resulting in a falsely high systolic pressure [48]. Other factors contributing to bias include sensor accuracy and the software/firmware logic [42]. Moreover, the cuff’s deflation rate, or ‘bleed rate’, is assumed to be consistent when the algorithm calculates the systolic and diastolic pressures [42], which may not hold in practice.

The WHO regularly publishes and updates guidelines for standard practices to ensure that healthcare providers are cognizant of calibration requirements and potential biases to provide quality healthcare [63]. The process of calibrating medical equipment is different for each device. Most devices have built-in mechanisms for calibration. Manufacturers also advise regular maintenance based on the rate of utilization. Automatically calibrated devices can be used within predefined tolerances, beyond which they should be returned to the manufacturer for technical inspection or disposed of.

#### 5.1.2. Consumables of Blood Pressure Measurement Devices

All BP devices have consumable components that require regular replacement. This includes batteries, rubber tubing, hoses, fittings, and cuffs [13]. For example, since the BP device consumes variable power throughout the inflation-deflation cycle, weak batteries may result in erroneous BP values. Worn cuffs and rubber tubing with weak elasticity also impact the reported BP values. Consumable parts of BP devices should, therefore, be replaced according to the manufacturers’ recommended timelines.

### 5.2. Subject-Specific Biases

BP values can significantly fluctuate across subjects [64]. In the following section, we list some of the most important sources of subject-specific bias.

#### 5.2.1. Demographic Features

Demographic features, such as sex, age, race, and genetic background, influence BP measurements due to variations in physiology and anatomy.

Sex is a prominent factor that influences BP [65,66]. Generally, males tend to have higher BP levels, which can be associated with differences in hormonal activities and anatomical differences between male and female bodies [66,67]. A notable study involving 32,833 individuals ranging from 5 to 98 years (54% women) was conducted over four decades [68]. The study showed that women exhibited a steeper increase in BP measurements compared to men, starting as early as the third decade of life and continuing throughout their lifespan [68]. Table 3 presents a compilation of studies reporting BP values based on sex.

Age is another significant demographic feature. Arterial stiffness is known to increase with age, leading to reduced arterial compliance and an increase in pulse pressure [57]. Elderly individuals may experience systolic hypertension, characterized by elevated SBP without a corresponding increase in DBP. This condition, commonly known as “pseudohypertension”, has been linked to a decline in arterial distensibility, which can lead to bias and inaccurately high BP readings, as the external cuff pressure on the artery is reduced [57]. Carrico et al. examined the relationship between age, sex, and BP in a group comprising 965 men and 1114 women [87]. Their findings indicated that BP values generally increase with age but decrease again after approximately 70 years of age. In most age groups, BP is higher in males than in females, but both sexes have similar BP until their teenage years. However, after about 70 years of age, females’ SBP values surpass those of males, whereas their DBP values remain approximately the same.

There has also been significant interest in examining health measures across racial groups. Numerous studies have examined the relationship between BP and race [88,89]. These studies have found that specific diseases are more common in certain racial groups, suggesting a potential link between race and hypertension prevalence [90,91]. For instance, Staessen et al. [92] showed that Asian populations have a higher rate of non-dipping compared to European populations, indicating possible variations in BP patterns among different races. Furthermore, individuals of African American descent have been observed to have higher rates of hypertension, cardiovascular, and cerebrovascular morbidity and mortality compared to those of European descent [93]. This suggests that race (or, more accurately, genetic factors) may play a significant role in hypertension susceptibility. Another possible explanation is that the experiences of some racial or ethnic groups, including but not limited to the medical care that these groups receive, affect their BP. Most likely, multiple factors contribute to the higher prevalence of hypertension in African Americans, including genetic, socioeconomic, systemic, and other factors. Table 4 summarizes the group of studies that have compared BP values based on race.

#### 5.2.2. Subject-Specific Factors

Height and weight are two essential factors influencing BP values. Studies have shown that taller individuals tend to have lower SBP and higher DBP compared to shorter individuals [85]. Body mass index (BMI)—weight divided by the squared height (kg/m^2^)—can be used as a compound factor for BP assessment [94,95]. Table 5 presents the findings from various studies that have reported BP values based on BMI, showing an overall positive correlation between BP and BMI [96].

#### 5.2.3. Subject-Specific Background Medical Conditions

Accurate interpretation of BP measurements should consider patient-specific conditions and comorbid factors, which can directly or indirectly influence BP readings. Often, the inherent biological rhythms in the disease process and their potential clinical implications are overlooked or inadequately considered when treating hypertensive patients [53]. In the following section, we discuss several situations where these considerations play a significant role.

Healthy individuals typically show BP variations throughout the day, rising during the day and dropping at night [115]. Most people have nighttime BP readings 10–20% lower than those in the daytime. However, some experience a non-dipping pattern, where nighttime BP drops by less than 10% [116]. About 25% of hypertensive individuals with unknown causes exhibit this non-dipping pattern [117].

The *“white-coat effect”*, caused by the medical environment and presence of a physician, can elevate a patient’s clinic BP [48]. This effect often results in higher SBP and DBP compared to baseline ambulatory BP [58]. Table 6 summarizes studies comparing BP measurements in settings like the home and office.

Pregnancy-related hemodynamic changes can affect BP readings. Although many automated BP device types exist, few are calibrated for pregnant women, including those with hypertensive disorders [14]. Nearly 10% of pregnant women face high BP, risking both the fetus(es) and the mother. Those with gestational diabetes or preeclampsia are especially at risk [54,120,121].

Obesity correlates with high BP in both children and adults. For each BMI unit increase, SBP and DBP rise by 0.56 and 0.54 mmHg, respectively, in obese children [94]. Table 7 presents studies examining the impact of obesity on children’s BP values.

#### 5.2.4. Eating, Drinking, and Smoking

Eating, drinking, and smoking habits can significantly influence BP values [105,122,123]. Studies indicate that eating has both short-term and long-term effects on BP levels [124].

Postprandial (post-meal) BP initially rises due to increased activity but later drops, particularly due to decreased total peripheral resistance from visceral vasodilation [125]. This reduction is pronounced in elderly, hypertensive individuals and those with autonomic failure [126]. High-carbohydrate meals cause a larger decrease in BP compared to high-fat ones. Although the exact mechanism for post-meal hypotension remains unclear, impacting factors may include impaired baroreceptor reflexes, insulin-induced vasodilation, and the release of vasodilatory gastrointestinal polypeptides [127]. This BP decrease peaks around 1 hour post-meal and can last over 2 hours, influencing diurnal BP changes, especially in elderly subjects with hypertension [125]. Furthermore, BP effects from alcohol, caffeine, and nicotine vary by dose and individual [58].

For the long-term impacts of eating habits on BP, guidelines primarily recommend lifestyle and dietary changes to manage hypertension, with an emphasis on reducing salt intake. Adding potassium-rich foods like nuts, fruits, and vegetables to the diet can enhance BP control [128]. Table 8 summarizes studies on the impacts of eating, drinking, or smoking on BP.

#### 5.2.5. Circadian Rhythm

The circadian clock significantly affects CVD risk factors like heart rate and BP [134]. It has been linked to two mechanisms: the central clock in the hypothalamic suprachiasmatic nucleus (SCN) and the peripheral clock in most body tissues and organs [135]. Environmental factors, like physical exercise, can also impact and align circadian rhythms, especially in skeletal muscle [136].

BP displays daily rhythmic fluctuations, peaking in the early morning and dipping around midnight. Boivin et al. [137] examined BP changes in the morning and evening among 52 controlled hypertensive patients. BP was measured six times in both the morning and evening, three times before and after resting. Each session was nine minutes with a minute between measurements and five minutes of rest. The study found significant BP differences between morning and evening of around 5 mmHg for DBP and 8 mmHg for SBP.

Psychological and physical activities also contribute to BP fluctuations, with higher values commonly observed during work hours and lower values at home. While several neurohormonal systems follow a circadian rhythm with a morning peak, the sympathetic nervous system appears to be the primary determinant of these BP circadian variations. However, this principle can be reversed for individuals with specific job roles, such as shift workers [53].

Table 9 provides a comparative analysis of studies exploring the effects of the circadian clock on BP values.

### 5.3. Biases Related to the Acquisition Session

There are specific guidelines for BP measurement, violations of which can lead to inaccurate readings. Here, we explore the often-overlooked factors during BP acquisition that can introduce bias and compromise measurement reliability.

#### 5.3.1. Seasonal Variations and Ambient Temperature

Seasonal BP fluctuations, with peaks in winter and troughs in summer, have been reported in both home and clinic measurements [138,139]. Although the direct link between BP and ambient temperature has not been conclusively established, consistent seasonal patterns suggest a correlation [140]. Yang et al.’s research on 23,040 individuals in China found BP values to be highest in winter (December–February) and lowest in summer (July–August) [141]. Table 10 summarizes studies comparing BP values by environmental temperature. The short-term impact of temperature on BP underscores the importance of protective measures in colder weather to maintain stable BP and reduce BP-related disease risks [104].

#### 5.3.2. Cuff Position

**Figure 4:**
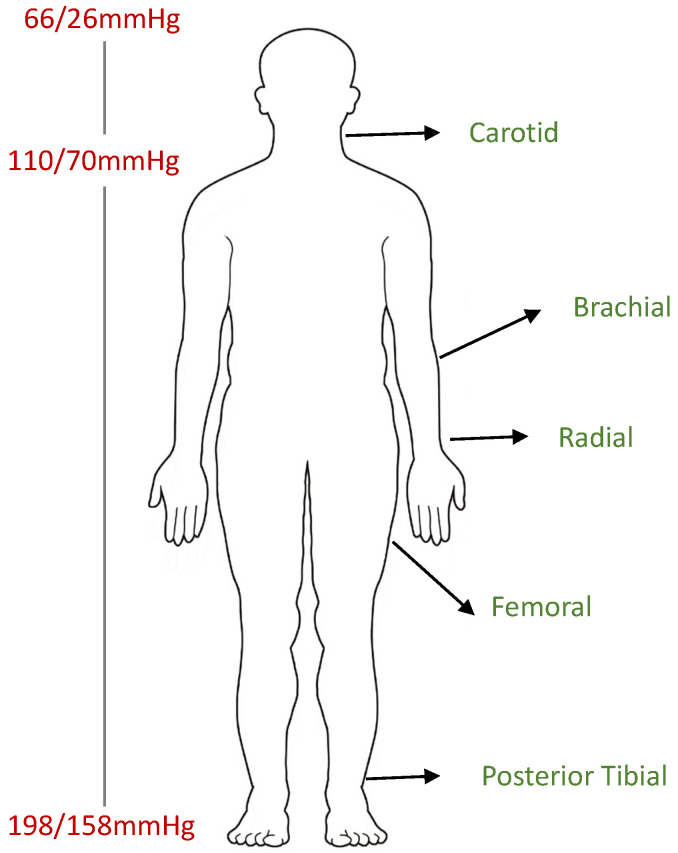
Schematic showing blood pressure variations across arterial sites (carotid, brachial, radial, femoral, posterior tibial) influenced by distance from the heart, artery type, and gravity in a standing position [145,146].

The brachial artery is commonly used for BP measurement [57,144]. While wrist and finger monitors have become popular, SBP and DBP values differ across the arterial tree [48]. Figure 4 depicts the influence of various body points on BP as blood flows through different arteries when upright. Table 11 summarizes studies comparing BP across different body points.

#### 5.3.3. Body Position

Body position significantly affects BP readings. Guidelines suggest measuring BP in a seated position with back support [59]. Research indicates BP values are higher when seated compared to lying down [81,102,106]. Specifically, sitting upright can increase DBP by up to 6.5 mmHg compared to leaning back [48]. For accurate measurements, the BP cuff should be at the level of the patient’s right atrium, regardless of position [57]. Table 12 summarizes studies on BP values for various body positions.

#### 5.3.4. Arm Position

Proper arm positioning during BP measurements is vital, with the arm ideally supported at heart level on a flat surface [48,102,152]. Shifting the arm from horizontal to vertical can raise the pressure by 5–6 mmHg due to hydrostatic changes [48]. Mariotti et al. explored the effects of arm positioning and postural hypotension during BP assessments [153], finding that incorrect arm positioning while standing resulted in overestimated BP readings. Table 13 summarizes studies on BP values considering different arm positions.

#### 5.3.5. Leg Position

Proper leg positioning during BP measurements is crucial according to guidelines [155]. Studies show that crossed-leg positions yield higher BP readings compared to uncrossed legs or sitting with feet flat [58,156]. Table 14 provides a summary of studies on BP values with varying leg positions, emphasizing the importance of standardized leg positioning for accurate measurements.

#### 5.3.6. Left vs. Right Arm

Clinical settings often show BP reading differences between the left and right arms [158]. Guidelines suggest measuring BP in both arms on the first visit, using the arm with higher readings thereafter [57]. Conditions like coarctation of the aorta or upper-extremity arterial obstruction can cause significant BP variations between arms [57]. Table 15 summarizes studies comparing BP measurements in both arms.

#### 5.3.7. Cuff Size and Tightness

Using the correct cuff size is vital for accurate BP measurement [160]. Small cuffs can overestimate pressure, which is a frequent error [48,161]. Larger cuffs tend to report lower BP values [58]. For children, cuff size selection is challenging. The BHS suggests three sizes based on arm circumference [48]: 4 × 13 cm, 8 × 18 cm, and 12 × 35 cm (adult cuff).

In obese individuals, selecting the right cuff size is essential to ensure that the brachial artery is accurately compressed, yielding reliable BP readings [57]. Many obese individuals have tronco-conical arms, further complicating accurate BP measurement [161]. For these patients, conical cuffs can offer more accurate BP readings [57]. Table 16 presents studies exploring the influence of cuff size on BP readings.

When measuring BP, the cuff should be securely placed around the upper arm without any clothing interference, ensuring even snugness from top to bottom. The tightness can be gauged by fitting one finger easily and two fingers with comfort between the cuff’s top and bottom. Achieving the correct cuff tightness is vital for reliable and consistent BP readings [57].

#### 5.3.8. Rest Period before Measuring BP

Studies have investigated the effects of rest durations before taking BP measurements. Their findings indicate that not resting sufficiently before a measurement can lead to elevated SBP and DBP readings. A rest period ranging from 10 to 16 min showed a modest decrease in SBP and a slight drop in DBP [163,164]. However, the exact duration of rest needed to compensate for the effects of prior physical activity remains uncertain. More research is needed to specify the ideal rest period for precise BP readings [58]. A summary of studies exploring the influence of the duration of rest on BP measurements can be found in Table 17.

#### 5.3.9. Number of Measurements

For many individuals, the first BP reading taken in a clinical setting tends to be higher than the readings that follow. Research has explored this pattern by conducting three consecutive BP measurements [57]. Findings showed that when only the first measurement was considered, approximately 35% of adults exhibited an SBP range of 140–159 mmHg and a DBP range of 90–99 mmHg. However, when the average of all three measurements was taken into account, most participants registered SBP/DBP values below the 140/90 mmHg benchmark [57]. Therefore, relying exclusively on the initial reading can result in over-diagnosing hypertension, emphasizing the necessity of multiple readings to ensure an accurate diagnosis [165].

#### 5.3.10. Clothing

Healthcare professionals are advised to measure BP by fully exposing the cuff on the upper arm. However, it is a common practice to measure BP by rolling up the sleeve or placing the cuff over the sleeves. Table 18 presents the findings from various studies that have examined the impact of clothing on BP values, demonstrating a bias in the BP readings due to clothing.

## 6. Cuff-Less Blood Pressure Technologies

Significant efforts have been made to develop novel and versatile methods for measuring BP. Traditional BP measurement techniques involving cuffs have several inherent limitations [43], such as (1) time-consuming procedures; (2) patient discomfort due to frequent cuff inflation, as seen in ABPM devices used for recording BP during the day and night [166]; (3) impracticality for continuous BP measurement in specific medical settings such as heart surgical units and acute burns cases; and (4) the necessity of allowing sufficient time between successive BP measurements to enable the blood vessels beneath the cuff to return to their baseline state and to prevent vessel collapse due to cuff pressure. To address these limitations, cuff-less BP measurement methods have been introduced, which can be categorized into three groups [11,26,167]: tonometry, volume clamps, and pulse-wave velocity. These innovative approaches offer the potential to overcome the drawbacks associated with traditional cuff-based BP measurement techniques, paving the way for more efficient and patient-friendly BP monitoring solutions.

The pulse-wave velocity (PWV) method of measuring BP was invented by Moens and Korteweg [40]. They defined a fundamental relationship between the vascular elasticity and pulse-wave velocity in the artery, known as the Moens–Korteweg equation [43,168]:

(2) $$\mathrm{PWV}=\sqrt{\frac{E\cdot h}{\rho \cdot D}}$$

where *h* represents the thickness of the vessel wall, ρ is the blood density, and *D* is the vessel’s inner diameter. The parameter *E* denotes Young’s modulus of elasticity, which indicates the vessel wall’s elasticity. Geddes empirically defined *E* as follows [43]:

(3) $$E=E_{0}e^{\alpha \cdot \mathrm{BP}}$$

where $E_{0}$ represents the modulus of elasticity at a pressure of 0 mmHg, α is a constant related to the vessel (typically ranging from 0.016 to 0.018 mmHg^−1^), *e* is the Euler number, and BP is the blood pressure. The Moens–Korteweg equation and Geddes’ formulation are fundamental in enabling the utilization of the PWV as a valuable and non-invasive method for assessing BP and vascular elasticity. Combining (2) and (3) leads to a compact relation between BP and PWV:

(4) $$\mathrm{BP}=\frac{1}{\alpha }\ln\frac{\rho \cdot D\cdot {\mathrm{PWV}}^{2}}{h\cdot E_{0}}$$

Several methods are used to measure the PWV. The pulse transit time (PTT) is the most well-known indirect method of calculating the PWV [9]. The relationship between the PTT and PWV is defined as follows [43]:

(5) $$\mathrm{PWV}=\frac{d}{\mathrm{PTT}}$$

where *d* is the distance between the heart and a specific location where the blood flows and PTT is the time it takes for the blood pulse to propagate the distance *d*. PTT is calculated using different sensors and bio-signals, including [169] photoplethysmography (PPG) and the output signal of the Hall sensor [170]; the PPG signal and the modulated magnetic signature of the blood [171]; PPG and ballistocardiography [172]; PPG and impedance plethysmography [173]; PPG and electrocardiography [174]; or one or two PPG recordings [175].

The parameter *d* is challenging to accurately determine in practice. Therefore, PWV and subsequently BP in (5) are difficult to accurately determine. To address this challenge, cuff-less BP monitoring devices are increasingly integrating artificial intelligence (AI) algorithms to learn crucial and complex features of the cardiovascular system [11,16]. Ongoing research is focused on exploring how to extract the most relevant features from signals like the PTT to model the cardiovascular system and effectively predict BP values. However, it is essential to acknowledge that these studies are still in the prototype stage and must be validated in large cohorts to meet medical standards before being used in clinical settings [11]. Nonetheless, advances in this domain show promise for enhancing BP monitoring and improving patient care.

When developing cuff-less methods, cuff-based BP databases are typically used as references to demonstrate their “substantial equivalency” with existing technology, as required for regulatory clearance like FDA 510(k) premarket approval [176,177,178,179]. If standard requirements are not met during cuff-based BP acquisition, the calibration models for new technologies will be trained with biased recordings. This affects the long-term reliability of these technologies, potentially causing deviations between predicted and actual BP values. It is essential to address these biases for accurate measurements with cuff-less technologies, warranting further research to enhance their performance and reliability.

## 7. Future Perspectives: Using Machine Learning for Bias Removal and Individualized BP-Level Risk Assessment

In our study, we investigated BP and various measurement- and subject-related factors that impact the accurate collection and interpretation of BP. However, given the vast BP literature, it is unfeasible to comprehensively examine every potential source of bias. It is important to note that as of 4 February 2024, PubMed alone reports 687,705 instances of the keyword “blood pressure” in its databases (obtained using the Entrez Direct (EDirect) Linux tools from the NCBI [180]). In addition, there are billions of BP records in electronic health records (EHRs) worldwide. The one-by-one investigation of these rich resources is infeasible. We propose that future research can leverage machine learning and natural language processing techniques (especially the recent advances in large language models) to mine these massive data- and literature-driven resources in a more systematic manner. This could result in more conclusive findings regarding subject-specific BP-related risk factors across different demographic groups. Open-source codes for BP-related studies are another requirement. Schwenck et al. recently released a versatile open-source BP analysis and visualization toolkit in R [181]. Beyond visualization tools, rigorous statistical frameworks and models are required for building machine learning pipelines. In the sequel to this paper, we propose a stochastic framework that can be used in future research to use machine learning algorithms in BP-related studies.

The problem of BP measurement can be formulated as follows:

(6) $$y_{k}={\mathrm{BP}}_{k}+e_{k}$$

where $y_{k}$ represents the reported BP values in different trials/sessions, ${\mathrm{BP}}_{k}$ is the ‘true’ BP value, and $e_{k}$ denotes the total measurement errors attributed to nonstandard devices or measurement errors. Both the BP and its error can be considered random variables, with presumed (time-variant) distributions. With this data model, the problem of accurate BP measurement and bias removal can be formulated as a classical estimation problem that could be addressed using standard techniques such as least squares, maximum likelihood, or Bayesian estimation, where the latter two benefit from prior distributions of the BP and measurement errors.

$e_{k}$ is device- and technology-dependent. Complying with standard BP measurement techniques and averaging over multiple trials (k=1,…,K) can mitigate the impact of this term, resulting in a more accurate measurement of BP.

Even with accurate measurements ($e_{k}\approx 0$), ${\mathrm{BP}}_{k}$ is a random variable, which fluctuates over time and across trials. A comprehensive machine learning framework should benefit from the demographic-specific distributions of ${\mathrm{BP}}_{k}$, namely:

(7) $$f({\mathrm{BP}}_{k}|p)$$

which is the conditional distribution of the BP (SBP, DBP, or both) given all the demographic and comorbid factors parameterized by the vector p (sex, race, age, background medical conditions, comorbidities, etc.).

**Figure 5:**
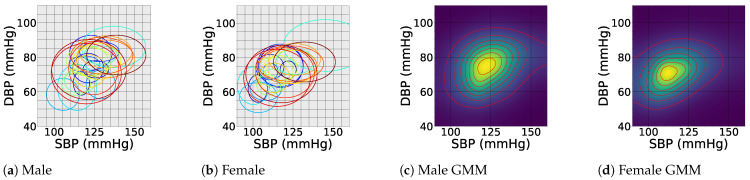
Comparison of male (**a**) vs. female (**b**) blood pressure values from various studies. Each ellipse represents a study, centered on the mean SBP and DBP, with horizontal and vertical radii representing the corresponding standard deviations. Gaussian mixture model (GMM) distributions are fitted over these reports in (**c**) and (**d**). For further clarification, each ellipse is assigned a specific color.

**Figure 6:**
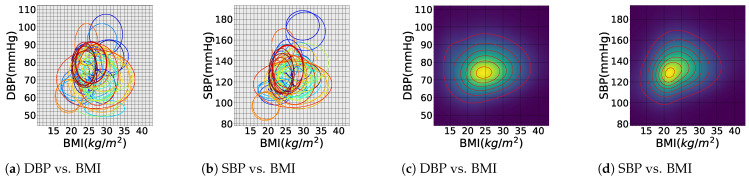
Comparison of blood pressure values by BMI from various studies. Each ellipse represents a study, centered on mean BMI and BP, with horizontal and vertical radii representing their respective standard deviations. For further clarification, each ellipse is assigned a specific color. Gaussian mixture model (GMM) distributions are fitted over these reports in (**c**) and (**d**).

We propose that our current survey of the BP literature—and more systematic future surveys that could benefit from massive EHR registries combined with NLP technologies for BP literature analysis—can be used to estimate $f({\mathrm{BP}}_{k}|p)$ across different demographic factors. As proof of concept, graphical representations of the BP data on sex and BMI from Table 3 and Table 5 are shown in Figure 5 and Figure 6, respectively. In Figure 5, each ellipse corresponds to an individual study from the studied literature, where each ellipse is centered at the mean SBP and DBP, with the horizontal and vertical radii of the ellipses representing the standard deviation of the reported SBP and DBP, respectively. Similar elliptical shapes can be constructed from massive EHR data while accounting for individualized and demographic factors. For multi-site studies, this information can be used to construct multi-modal distributions of $f({\mathrm{BP}}_{k}|p)$. For instance, assuming that the reported BP values follow a normal distribution (which is an accurate assumption for population-wide studies), the multi-site data can be effectively modeled using Gaussian mixture models (GMMs) to capture underlying clusters or sub-populations [182]. The heatmaps in Figure 5a,b and Figure 6c,d correspond to GMM distributions fitted over the ensemble of the studied BP reports for sex and BMI.

An interesting observation from these figures is that while the literature on BP merely reports the mean and standard deviation of the SBP and DBP, as well as the pulse pressure, there is a trend of correlation between the SBP and DBP, which has not been studied in the BP literature. BP data from EHRs can additionally be used to identify the correlations between SBP and DBP. In recent research, we have calculated the correlation coefficient and other statistical properties of the SBP–DPB distribution on a large cohort of 75 million patient encounters [183].

**Figure 7:**
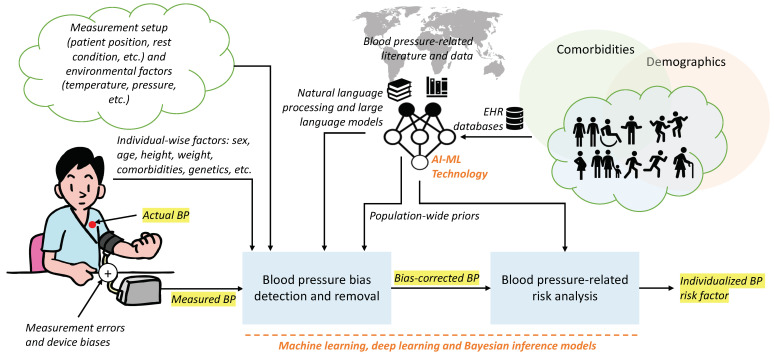
A future perspective of blood pressure (BP) monitoring. AI-assisted and ML-based technologies are anticipated to integrate into future generations of BP monitoring technologies. Large electronic health record (EHR) data can be leveraged to extract population-wide norms of BP, accounting for variations due to biological sex, age, and race (from a genetic variability standpoint) [183]. Natural language processing and large language models can assist in analyzing clinical literature and physicians’ notes from EHR data [187]. These data resources can be combined using an ML framework to detect and correct BP measurement biases and provide individualized BP-related risk factors.

Future research can integrate these distributions and the data model in (6) to provide Bayesian estimates of BP on a subject-specific basis. These distributions can also be integrated with EHR data and clinical outcomes to train machine learning technologies that quantify the risk of hypertension and BP-related complications of individuals across different demographics (see Figure 7), resulting in a more accurate assessment of BP-based diagnoses [184]. With such technology, we anticipate being able to express a patient’s BP-associated risk using terms such as *“A 55-yr-old Hispanic female with a body mass index of 34.2, a consistent in-clinic cuff-based systolic BP > 129 mmHg, a diastolic BP > 85 mmHg while sitting, and a history of diabetes is 15% at risk of stroke in the next 12 years (p-value < 0.05)”*.

We envision that this risk index will be integrated with well-established risk scores, such as the Framingham Risk Score (which estimates a person’s 10-year risk of developing cardiovascular disease, considering factors like sex, age, cholesterol levels, and BP) [185] or the HEART Score [186] (which is used in emergency settings to assess the risk of major adverse cardiac events in patients presenting with chest pain based on criteria such as medical history, ECG results, etc.).

BP measurement devices require maintenance and regular calibration to identify and reduce measurement uncertainties to an acceptable level. The identification and correction of systematic errors are performed using reference instruments that have been well maintained and calibrated by medical instrumentation experts. The process of calibrating medical equipment varies for each device. While integrating AI and ML techniques into current BP devices may not directly reduce systematic errors related to the primary BP measurement unit, envisioning the future of BP technologies suggests promising possibilities for minimizing such errors. In the future, BP devices could be equipped with integrated technologies designed to significantly enhance accuracy and address systematic errors. This could involve integrating online technologies that compare an individual’s BP with their historical records, clinical data, and broader population-wide information.

Integrating AI and ML techniques into the next generation of BP devices would require substantial computing capabilities. A possible technological advancement is to implement low-computation versions of machine and deep learning models in the software/firmware of BP devices or to link them with mobile phones to process and transmit the BP data to cloud platforms. The former would require more powerful processors and/or tensor flow units (TPUs) in BP devices, enabling the implementation of recent technologies such as TinyML and TensorFlow Lite on edge devices. Pre-trained models may also be integrated into the BP device’s software or released as mobile applications for efficient and accurate BP assessment without extensive computing power within the device itself.

Incorporating AI and ML into BP measurement technologies also promises enhanced accuracy and personalized monitoring. By utilizing large BP record databases and state-of-the-art machine and deep learning models, these algorithms aim to reduce biases, leading to precise BP monitoring and tailored cardiovascular risk assessments. These models can be provided with inputs like measurement setup (patient position, rest condition), environmental factors, and individual-specific details (sex, age, BMI, comorbidities, etc.), complemented by population-wide data. AI/ML models can be trained to utilize these inputs to improve precision in BP measurements and risk assessment.

## 8. Conclusions

BP is a crucial vital sign for monitoring health and making clinical decisions. In recent years, the adoption of cuff-based BP technologies has surged, primarily driven by the growing popularity of ambulatory and home-based BP monitoring, as well as its widespread use in medical centers and hospitals. Accurate BP values are essential, as discrepancies between reported and actual BP measurements can lead to misinterpretation and mistreatment. In this study, we highlighted the notion of “bias” and the factors that may result in bias in cuff-based BP monitoring across various social groups. This survey demonstrated that reported BP values can be diverse due to individualized and demographic factors or inaccurate due to a failure to adhere to BP measurement standards. Given these limitations, the development of a new generation of cuff-based BP devices supported by artificial intelligence (AI) and machine learning (ML) techniques is in high demand. Integrating AI and ML techniques into these devices is a promising approach to identifying and correcting bias, as well as customizing normal and abnormal BP ranges on an individualized level, thereby improving the accuracy of BP measurements and clinical decision making. In conclusion, addressing the issue of bias in cuff-based BP devices is essential for advancing patient care and ensuring reliable BP data for medical decision making. Developing ML-based devices that can detect and correct bias will undoubtedly be a valuable contribution to the field, enhancing the overall effectiveness and reliability of BP monitoring systems. By focusing on improving BP monitoring precision, we can significantly improve patient outcomes.

## Figures and Tables

**Table 1:** Blood pressure norms based on the health status of an adult [37].

BP Category	SBP (mmHg)		DBP (mmHg)
**Normal BP**	<120	AND	<80
**Elevated**	120–129	AND	<80
**High BP (Hypertension) Stage1**	130–139	OR	80–89
**High BP (Hypertension) Stage2**	≥140	OR	≥90

**Table 2:** AHA recommendations for the appropriate cuff sizes for patients [42].

Cuff	Arm Circumference (cm)	Bladder Width (cm)	Bladder Length (cm)
**Newborn**	<6	3	6
**Infant**	6–15	5	15
**Child**	16–21	8	21
**Small Adult**	22–26	10	24
**Adult**	27–34	13	30
**Large Adult**	35–52	20	42
**Adult Thigh**	45–52	20	42

**Table 3:** The results of studies reporting blood pressure values based on sex. N is the number of patients. Summaries of SBP and DBP include mean ± standard deviation.

Ref.	Total N	Male			Female		
		**N**	**SBP**	**DBP**	**N**	**SBP**	**DBP**
[69]	20	10	126.0 ± 8.0	73.0 ± 5.0	10	122.0 ± 5.0	73.0 ± 5.0
[69]	26	13	117.0 ± 5.0	65.0 ± 7.0	13	103.0 ± 6.0	62.0 ± 8.0
[70]	37	22	121.2 ± 9.7	73.7 ± 8.5	15	117.4 ± 13.9	74.8 ± 12.2
[71]	39	24	122.9 ± 13.2	82.6 ± 10.1	15	110.5 ± 8.8	74.5 ± 7.3
[72]	40	20	128.2 ± 12.3	83.3 ± 5.8	20	117.0 ± 14.4	75.5 ± 12.3
[73]	45	23	119.0 ± 9.5	76.0 ± 4.7	22	111.0 ± 4.6	72.0 ± 4.6
[74]	55	26	129.1 ± 9.1	64.2 ± 8.3	29	108.0 ± 9.8	61.7 ± 6.7
[75]	92	55	105.6 ± 10.3	58.5 ± 9.3	37	103.4 ± 11.8	56.9 ± 8.9
[75]	107	42	114.3 ± 12.2	62.5 ± 13.3	65	100.3 ± 10.0	63.6 ± 10.9
[76]	122	52	137.0 ± 20.0	86.0 ± 12.0	70	145.0 ± 26.0	87.0 ± 15.0
[77]	141	117	128.8 ± 10.4	81.3 ± 5.3	24	126.0 ± 11.8	77.6 ± 7.4
[78]	312	142	116.3 ± 9.9	66.4 ± 7.1	170	112.3 ± 8.3	66.5 ± 6.8
[78]	351	184	113.7 ± 9.0	64.5 ± 6.6	167	109.8 ± 7.5	64.1 ± 5.9
[79]	806	237	120.6 ± 12.9	77.9 ± 8.7	569	112.7 ± 12.3	71.7 ± 8.4
[80]	1030	614	123.3 ± 12.3	77.3 ± 8.2	416	117.1 ± 10.6	73.9 ± 7.1
[81]	1298	638	127.4 ± 14.0	77.7 ± 10.5	660	124.4 ± 15.7	74.5 ± 9.7
[82]	1378	664	122.0 ± 10.5	72.0 ± 9.4	714	113.0 ± 9.9	68.2 ± 8.6
[82]	1534	767	132.0 ± 16.4	83.1 ± 9.3	767	126.0 ± 16.1	78.9 ± 9.2
[83]	2105	945	132.0 ± 18.0	79.0 ± 11.0	1160	122.0 ± 18.0	75.0 ± 10.0
[84]	2442	1577	129.7 ± 19.2	80.9 ± 10.6	865	123.2 ± 20.9	76.5 ± 10.3
[85]	2849	1505	124.6 ± 15.5	73.8 ± 15.5	1344	120.0 ± 18.3	71.2 ± 14.6
[85]	3654	1915	120.0 ± 21.8	70.5 ± 17.5	1739	115.0 ± 20.8	68.2 ± 16.6
[85]	6485	3379	121.7 ± 23.2	72.9 ± 17.4	3106	117.8 ± 22.2	70.4 ± 16.7
[86]	33,599	19,704	138.7 ± 18.4	81.3 ± 11.5	13895	132.1 ± 19.3	77.4 ± 11.6

**Table 4:** Summary of results of studies reporting blood pressure values across races. N is the number of patients. Summaries of SBP and DBP include mean ± standard deviation.

Ref.	N	Race	SBP	DBP
[75]	199	Black	105.7 ± 10.9	63.2 ± 11.9
		White	104.7 ± 10.9	57.9 ± 9.1
[93]	245	White hypertensive	145.0 ± 18.3	92.0 ± 10.7
		Black hypertensive	142.0 ± 14.9	93.0 ± 10.8
[78]	663	European American	111.8 ± 8.3	64.3 ± 6.2
		African American	114.1 ± 9.0	66.4 ± 6.9
[85]	6503	Non-Hispanic Black	122.4 ± 16.9	72.5 ± 21.3
		Mexican American	117.6 ± 30.2	69.4 ± 24.1
[85]	9334	Non-Hispanic White	119.8 ± 32.2	71.7 ± 24.1
		Non-Hispanic Black	122.4 ± 16.9	72.5 ± 21.3
[85]	10,139	Non-Hispanic White	119.8 ± 32.2	71.7 ± 24.1
		Mexican American	117.6 ± 30.2	69.4 ± 24.1

**Table 5:** The results of studies reporting blood pressure values based on BMI.

Ref.	N	BMI	SBP	DBP
[97]	13	30.7 ± 4.2	124.7 ± 13.0	82.4 ± 10.1
[98]	17	24.3 ± 2.4	115.4 ± 6.2	68.5 ± 5.4
[99]	21	23.9 ± 3.3	115.4 ± 13.5	71.2 ± 9.4
[72]	40	23.6 ± 3.5	122.6 ± 14.4	79.4 ± 10.3
[100]	45	29.8 ± 4.7	174.0 ± 14.1	95.8 ± 11.5
[73]	45	22.6 ± 2.6	115.0 ± 6.7	74.0 ± 6.7
[101]	50	28.6 ± 3.9	133.9 ± 12.3	66.4 ± 9.7
[102]	57	25.7 ± 4.4	135.7 ± 24.8	79.5 ± 9.7
[100]	70	30.6 ± 5.6	168.3 ± 18.4	83.4 ± 9.4
[103]	88	22.0 ± 4.4	108.0 ± 10.0	65.0 ± 9.0
[104]	100	23.7 ± 2.9	132.9 ± 16.5	80.0 ± 10.4
[105]	165	21.3 ± 4.1	112.0 ± 10.0	67.0 ± 9.0
[103]	194	26.0 ± 5.0	120.3 ± 15.8	76.4 ± 11.3
[106]	280	28.7 ± 4.2	143.8 ± 14.3	92.4 ± 9.5
[107]	287	25.0 ± 3.9	139.2 ± 16.9	74.6 ± 12.0
[78]	312	24.0 ± 7.0	114.1 ± 9.0	66.4 ± 6.9
[78]	351	22.0 ± 5.0	111.8 ± 8.3	64.3 ± 6.2
[108]	389	29.4 ± 5.7	121.1 ± 16.3	53.8 ± 4.8
[109]	500	27.9 ± 5.3	123.0 ± 17.0	70.0 ± 11.0
[109]	599	28.1 ± 5.1	128.0 ± 18.0	72.0 ± 12.0
[81]	638	27.5 ± 3.5	127.4 ± 14.0	77.7 ± 10.5
[81]	660	27.3 ± 5.2	124.4 ± 15.7	74.5 ± 9.7
[109]	733	28.0 ± 5.2	122.0 ± 17.0	69.0 ± 11.0
[109]	735	28.2 ± 5.5	124.0 ± 18.0	71.0 ± 11.0
[79]	806	23.7 ± 3.0	115.0 ± 13.0	73.5 ± 8.9
[108]	833	27.5 ± 4.7	124.3 ± 9.5	68.5 ± 6.1
[108]	927	27.3 ± 5.6	117.1 ± 14.3	53.9 ± 4.6
[83]	945	26.1 ± 4.4	132.0 ± 18.0	79.0 ± 11.0
[108]	1030	30.8 ± 6.3	138.1 ± 18.6	71.9 ± 8.6
[83]	1160	25.7 ± 5.2	122.0 ± 18.0	75.0 ± 10.0
[81]	1298	27.4 ± 4.5	125.9 ± 14.9	76.1 ± 10.2
[85]	1344	30.0 ± 7.3	120.0 ± 18.3	71.2 ± 14.6
[82]	1378	23.4 ± 3.5	112.5 ± 10.1	70.0 ± 8.9
[85]	1505	27.1 ± 7.7	124.6 ± 15.5	73.8 ± 15.5
[82]	1534	26.5 ± 3.9	129.0 ± 16.2	81.0 ± 9.2
[108]	1559	28.8 ± 5.2	137.2 ± 16.4	71.8 ± 8.3
[85]	1739	28.6 ± 8.3	115.0 ± 20.8	68.2 ± 16.6
[85]	1915	27.7 ± 8.7	120.0 ± 21.8	70.5 ± 17.5
[110]	1937	19.2 ± 3.8	96.5 ± 13.3	60.6 ± 9.4
[110]	1968	19.5 ± 3.9	97.5 ± 13.2	61.3 ± 9.0
[83]	2105	25.9 ± 5.1	127.0 ± 19.0	77.0 ± 11.0
[111]	2423	24.3 ± 3.3	154.7 ± 16.2	90.1 ± 11.9
[84]	2442	24.9 ± 3.6	127.4 ± 20.1	79.4 ± 10.7
[85]	3106	26.9 ± 11.1	117.8 ± 22.2	70.4 ± 16.7
[85]	3379	27.5 ± 5.8	121.7 ± 23.2	72.9 ± 17.4
[107]	6887	25.7 ± 4.4	134.3 ± 20.2	79.6 ± 11.6
[107]	12,624	25.5 ± 4.4	131.9 ± 23.1	79.7 ± 11.9
[107]	17,921	25.6 ± 4.4	133.1 ± 22.4	79.9 ± 11.8
[112]	32,710	23.6 ± 3.3	123.6 ± 19.8	78.9 ± 12.4
[113]	417,907	23.8 ± 3.6	128.1 ± 19.0	76.1 ± 10.4
[114]	506,673	23.7 ± 3.4	131.0 ± 21.0	78.0 ± 11.0

**Table 6:** The results of studies reporting blood pressure values based on the measuring environment.

Ref.	N	Home			Clinic		
		**N**	**SBP**	**DBP**	**N**	**SBP**	**DBP**
[118]	454	199	144.0 ± 18.0	88.6 ± 10.0	255	160.0 ± 13.0	99.7 ± 4.0
[119]	574	287	125.7 ± 8.4	72.9 ± 8.6	287	139.2 ± 16.9	74.6 ± 12.0
[111]	4846	2423	152.4 ± 3.1	89.7 ± 9.3	2423	154.7 ± 16.2	90.1 ± 11.9
[107]	13,774	6887	127.3 ± 18.1	76.2 ± 9.9	6887	134.3 ± 20.2	79.6 ± 11.6
[107]	35,842	17,921	129.1 ± 18.6	76.9 ± 9.8	17921	133.1 ± 22.4	79.9 ± 11.8

**Table 7:** The results of studies investigating the effect of obesity on blood pressure values in the child population.

Ref.	BP	Sex	Obese Group		Non-Obese Group		Age (Years)
			**N**	**Mean ± SD**	**N**	**Mean ± SD**	
[94]	SBP	Boys	330	96.0 ± 13.3	331	90.0 ± 10.6	0.1–6.9
	SBP	Girls	253	95.0 ± 13.2	251	90.0 ± 11.5	
[94]	DBP	Boys	330	60.0 ± 10.7	331	60.0 ± 9.5	0.1–6.9
	DBP	Girls	253	60.0 ± 11.0	251	60.0 ± 10.0	
[110]	SBP	Boys	420	103.3 ± 14.8	1034	94.2 ± 11.8	6–11
	SBP	Girls	401	100.7 ± 14.1	1050	93.5 ± 11.8	
[110]	DBP	Boys	420	64.4 ± 9.8	1034	59.6 ± 8.7	6–11
	DBP	Girls	401	63.0 ± 9.3	1050	58.8 ± 9.2	
[95]	SBP	Boys	80	103.0 ± 13.0	143	98.0 ± 11.0	6–12
	SBP	Girls	28	99.0 ± 14.0	144	94.0 ± 11.0	
[95]	DBP	Boys	80	57.0 ± 9.0	143	55.0 ± 6.0	6–12
	DBP	Girls	28	55.0 ± 11.0	144	50.0 ± 6.0	

**Table 8:** Summarized results of studies investigating the impact of eating, drinking, or smoking on blood pressure values. “B.” denotes before and “A.” denotes after each activity.

Ref.	N	Conditions	SBP	DBP
[129]	11	B. drinking AF200 ^a^	120.0 ± 9.9	69.0 ± 3.3
		90 min. A. drinking AF200	123 ± 6.6	74.0 ± 3.3
[129]	11	B. drinking B350 ^b^	123.0 ± 6.6	71.0 ± 6.6
		90 min. A. drinking B350	123.0 ± 9.9	76.0 ± 13.2
[130]	12	B. drinking placebo	133.5 ± 14.1	86.4 ± 8.7
		A. drinking placebo	131.5 ± 11.8	82.9 ± 8.4
[130]	12	B. drinking C67 ^d^	127.6 ± 9.1	81.9 ± 6.7
		A. drinking C67	135.6 ± 10.1	84.7 ± 6.0
[130]	12	B. drinking C133 ^e^	126.9 ± 11.1	81.4 ± 7.7
		A. drinking C133	137.6 ± 14.1	86.5 ± 8.2
[130]	12	B. drinking C200 ^f^	127.5 ± 10.2	81.1 ± 5.5
		A. drinking C200	132.7 ± 10.7	83.5 ± 8.2
[131]	15	Pre-treatment of alcohol	120.0 ± 11.6	64.0 ± 7.7
		Post-treatment of alcohol	124.0 ± 15.5	69.0 ± 7.7
[131]	15	Pre-treatment of placebo	117.0 ± 7.7	64.0 ± 11.6
		Post-treatment of placebo	123.0 ± 7.7	71.0 ± 7.7
[99]	18	B. drinking alcohol ^c^	110.3 ± 12.0	80.0 ± 8.0
		A. drinking alcohol	109.5 ± 11.4	76.2 ± 7.1
[132]	22	B. drinking Noni juice	119.6 ± 8.3	77.0 ± 6.6
		A. drinking Noni juice	113.6 ± 8.5	72.0 ± 4.8
[132]	22	B. drinking chokeberry juice	125.6 ± 14	84.0 ± 9.8
		A. drinking chokeberry juice	124.3 ± 16.1	81.0 ± 9.9
[132]	22	B. consuming energy drink	119.2 ± 14.8	73.9 ± 8.4
		A. consuming energy drink	124.8 ± 14.1	84.8 ± 9.9
[132]	22	B. drinking water	124.3 ± 13.5	77.7 ± 9.2
		A. drinking water	124.0 ± 11.4	75.8 ± 8.0
[133]	35	B. drinking STING ^h^	123.0 ± 14.9	78.7 ± 10.5
		A. drinking STING	123.7 ± 14.5	78.2 ± 9.8
[70]	37	B. drinking 50 mL water	119.6 ± 11.5	74.1 ± 10.1
		A. drinking 50 mL water	122.5 ± 11.6	77.3 ± 7.7
[70]	37	B. drinking 500 mL water	116.9 ± 8.6	73.8 ± 10.0
		A. drinking 500 mL water	125.8 ± 8.8	76.8 ± 10.7
[71]	39	B. non-tobacco smoking	120.0 ± 13.5	78.9 ± 10.1
		65 min. A. non-tobacco smoking	125.8 ± 8.8	76.6 ± 6.9
[71]	39	B. tobacco smoking	118.6 ± 12.8	79.7 ± 9.2
		65 min. A. tobacco smoking	116.9 ± 12.4	80.0 ± 8.9
[72]	40	B. drinking 200 mL cold espresso	116.7 ± 9.7	75.3 ± 7.1
		A. drinking 200 mL cold espresso	120.0 ± 11.1	79.5 ± 9.1
[72]	40	B. drinking 200 mL filter coffee	118.2 ± 12.3	77.1 ± 8.5
		A. drinking 200 mL filter coffee	121.2 ± 10.6	79.1 ± 6.7
[72]	40	B. drinking 200 mL cold inst. coffee	116.7 ± 12.3	77.3 ± 8.5
		A. drinking 200 mL cold inst. coffee	121.3 ± 11.4	79.6 ± 7.3
[72]	40	B. drinking 200 mL hot inst. coffee	118.5 ± 10.5	78.2 ± 9.3
		A. drinking 200 mL hot inst. coffee	122.6 ± 11.8	80.2 ± 8.7
[133]	60	B. drinking STING ^g^	121.2 ± 14.3	77.4 ± 9.6
		A. drinking STING	126.5 ± 14.1	81.0 ± 9.0
[123]	72	Pre-treatment beverage of juice	117.0 ± 13.1	79.8 ± 10.1
		Post-treatment beverage of juice	125.9 ± 13.2	85.4 ± 9.6
[123]	72	Pre-treatment beverage of placebo	126.2 ± 19.2	83.5 ± 13.9
		Post-treatment beverage of placebo	130.7 ± 18.2	85.7 ± 12.9
[123]	72	Pre-treatment beverage of alcohol	116.9 ± 13.5	80.1 ± 8.7
		Post-treatment beverage of alcohol	113.2 ± 12.6	79.9 ± 9.7
[105]	194	B. water-pipe smoking	120.3 ± 15.8	76.4 ± 11.3
		15 min. A. water-pipe smoking	121.1 ± 16.1	77.1 ± 10.8

^a^ 200mL of alcohol-free beer; ^b^ 350mL of beer; ^c^ 2 glasses (2 × 125 mL) of red wine (12% ethanol); ^d^ 67mg of caffeine; ^e^ 133mg of caffeine; ^f^ 200mg of caffeine; ^g^ a single dose (500 mL) of energy drink; ^h^ 2 glasses (500 mL) of plain water.

**Table 9:** Results of studies investigating the impact of the circadian clock on blood pressure values.

Ref.	N	Mean Daytime BP	Mean Nighttime BP
		**SBP/DBP**	**SBP/DBP**
[93]	46	(149.0 ± 18.3)/(95.0 ± 10.7)	(132.0 ± 21.7)/(81.0 ± 13.5)
[93]	46	(145.0 ± 14.9)/(95.0 ± 11.5)	(136.0 ± 17.6)/(86.0 ± 11.5)
[106]	280	(144.7 ± 11.9)/(91.0 ± 8.6)	(128.2 ± 12.9)/(77.8 ± 9.0)
[78]	312	(119.5 ± 8.8)/(72.5 ± 6.6)	(108.7 ± 9.3)/(60.4 ± 7.2)
[78]	351	(117.7 ± 8.1)/(70.9 ± 6.4)	(105.9 ± 8.4)/(57.7 ± 6.1)
[107]	17,921	(129.3 ± 15.1)/(78.8 ± 9.3)	(112.9 ± 15.6)/(65.1 ± 9.6)

**Table 10:** Results of studies investigating the impact of ambient temperature on blood pressure values.

Ref.	N	Temp. (°C)	SBP	DBP
[140]	19	7.5 ± 0.7	118.0 ± 7.8	65.0 ± 6.1
[140]	20	14.8 ± 1.3	116.0 ± 6.3	64.0 ± 5.4
[140]	20	2.0 ± 0.4	121.0 ± 7.6	65.0 ± 6.3
[140]	20	−3.4 ± 3.0	125.0 ± 18.0	67.0 ± 5.8
[142]	39	25.0	117.0	65.0
[142]	39	17.6	117.0	66.0
[142]	39	22.7	122.0	65.0
[142]	39	21.6	119.0	65.0
[104]	100	15.7 ± 8.7	132.9 ± 16.5	80.0 ± 10.4
[143]	327	31.5 ± 1.0	133.7 ± 24.5	81.7 ± 15.4
[109]	500	25 ± 1	123.0 ± 17.0	70.0 ± 11.0
[109]	599	24 ± 1	128.0 ± 18.0	72.0 ± 12.0
[109]	733	26 ± 1	122.0 ± 17.0	69.0 ± 11.0
[109]	755	25 ± 1	124.0 ± 18.0	71.0 ± 11.0

**Table 11:** Summary of studies investigating the impact of body points on recorded blood pressure values.

Ref.	N	Measuring Place	SBP	DBP
[100]	45	Upper Arm	174.0 ± 14.1	95.8 ± 11.5
		Wrist	163.8 ± 25.4	94.4 ± 11.5
[100]	70	Upper Arm	168.3 ± 18.4	83.4 ± 9.4
		Wrist	159.2 ± 18.5	83.2 ± 10.5
[147]	250	Arm	127.7 ± 15.7	80.7 ± 11.2
		Leg	143.0 ± 22.2	75.7 ± 11.9

**Table 12:** Results of studies investigating blood pressure values for different positions of the subject during measurement.

Ref.	N	Body Position	SBP	DBP
[130]	12	Supine	116.2 ± 11.7	68.1 ± 6.6
		Upright	133.5 ± 14.1	86.4 ± 8.7
[102]	57	Sitting	135.7 ± 24.8	79.5 ± 9.7
		Supine	141.3 ± 25.5	84.6 ± 10.5
[148]	157	Sitting	102.8 ± 11.4	65.7 ± 8.2
		Standing	99.9 ± 10.2	66.0 ± 8.7
		Supine	107.9 ± 10.7	66.9 ± 9.6
		Supine; legs crossed	107.0 ± 8.6	66.7 ± 7.3
[149]	229	Supine	129.8 ± 27.5	72.5 ± 14.5
		Beach chair	114.6 ± 24.8	64.6 ± 11.2
[150]	245	Sitting	136.7 ± 21.9	86.0 ± 14
		Supine	135.5 ± 20.3	83.5 ± 12.5
[151]	250	Supine	139.3 ± 14.0	80.1 ± 9.1
		Fowler’s	138.1 ± 13.8	81.9 ± 9.4
		Sitting	137.2 ± 13.7	83.0 ± 9.6
[81]	1298	Sitting	125.9 ± 14.9	76.1 ± 10.2
		Supine	124.7 ± 14.1	71.7 ± 9.0

**Table 13:** Results of studies investigating the impact of arm position on blood pressure values.

Ref.	N	Arm Position	SBP	DBP
[102]	57	Arm high (at heart level)	137.4 ± 29.0	78.2 ± 14.4
		Arm low (on the bed)	142.1 ± 28.0	82.1 ± 13.4
[154]	69	Arm high (at heart level)	133.3 ± 20.7	77.7 ± 9.9
		Arm low (on chair arm-rest)	143.0 ± 19.9	88.6 ± 9.1

**Table 14:** Results of studies investigating the impact of leg position on blood pressure values.

Ref.	N	Leg Position	SBP	DBP
[155]	100	Uncrossed	146.5 ± 18.6	80.9 ± 11.2
		Crossed	155.6 ± 19.3	84.9 ± 11.6
[157]	238	Uncrossed	145.3 ± 20.3	86.4 ± 10.8
		Crossed	153.6 ± 20.2	92.1 ± 11.2

**Table 15:** Results of studies investigating the differences in blood pressure values between the right and left arms.

Ref.	N	Right Arm	Left Arm
[102]	57	SBP: 138.3 ± 29.2	SBP: 137.4 ± 29.0
		DBP: 77.8 ± 13.7	DBP:78.2 ± 14.4
[154]	69	SBP: 133.3 ± 20.7	SBP: 131.8 ± 19.1
		DBP: 77.7 ± 9.9	DBP: 78.0 ± 9.9
[159]	400	SBP: 131.2 ± 21.0	SBP: 129.4 ± 21.2
		DBP: 76.8 ± 11.9	DBP: 77.1 ± 12.6

**Table 16:** Results of studies investigating the effect of cuff size on blood pressure values.

Ref.	N	Cuff Size (cm)	SBP	DBP
[162]	130	13 × 36	125.1 ± 19.2	75.4 ± 12.4
		16 × 23	123.7 ± 19.7	74.4 ± 13.2
		13 × 23	127.2 ± 19.2	77.0 ± 12.8

**Table 17:** Results of studies investigating the effect of resting before measuring BP.

Ref.	N	Before Resting	After Resting	Resting Time
[137]	52	SBP: 127.9 ± 12.0	SBP: 121.5 ± 10.9	5 min
		DBP: 78.0 ± 8.7	DBP: 76.0 ± 9.0	

**Table 18:** Results of studies investigating the impact of wearing clothing on the arm during blood pressure measurements on reported values.

Ref.	N	Measuring Place	SBP	DBP
[77]	141	Sleeved	128.5 ± 10.6	80.7 ± 6.3
		Rolled sleeves	128.3 ± 11.1	80.9 ± 6.3
		Bare arm	128.4 ± 10.8	80.8 ± 6.0

## Data Availability

Not applicable.
